# Work-family and family-work conflicts amongst African nurses caring for patients with AIDS

**DOI:** 10.4102/curationis.v38i1.1436

**Published:** 2015-12-14

**Authors:** Lehlogonolo Makola, Solomon Mashegoane, Legesse K. Debusho

**Affiliations:** 1 Department of Psychology, University of Limpopo, South Africa; 2 Department of Statistics, University of South Africa, South Africa

## Abstract

**Background:**

South African nursing environments are marked by various incapacitating stressors. This study explores work-family (W-F) and family-work (F-W) conflicts as aspects of stress amongst nurses working with patients who have AIDS.

**Objectives:**

The study sought to determine the value of W-F and F-W conflicts as predictors of work and family satisfaction, as well as turnover intentions and the moderating role of supervisor and significant other support, amongst nurses caring for patients with AIDS in public hospitals within the Capricorn and Mopani districts, Limpopo Province.

**Methods:**

The study used a cross-sectional design, with data collected at one point only. Ninety-one nursing staff provided the data for the study by completing structured, self-administered surveys. Analysis involved computing correlations of all study variables. Thereafter, associated variables were used as predictors. In each predictive analysis, the nurses’ stress served as a control variable, W-F and F-W conflicts were the independent variables and significant others and supervisor supports were moderators. Interaction terms were derived from independent and moderator variables.

**Results:**

Although the findings of the study were not generally supportive of the hypotheses advanced, they nevertheless showed, amongst other findings, that F-W conflict predicted work satisfaction whilst W-F conflict predicted turnover intentions. Moreover, significant other support had a direct effect on family satisfaction whilst supervisor support moderated reports of W-F conflict and experiences of work satisfaction.

**Conclusions:**

The study showed that inter-role models that appear to be established in the context of developed societies require some further investigations in South Africa.

## Introduction

The work environment of South African nurses is characteristically stressful and produces a multiplicity of strains to role incumbents (Coetzee *et al.* 2013; Koekemoer & Mostert 2006; Pillay 2009; Van der Colff 2005). It is defined by extensive workloads, long working hours and unsatisfactory compensation. Nurses caring for patients with AIDS are particularly affected by stressors, causing a situation that affects their ability to engage in ethical practices and maintaining a therapeutic relationship (De Villiers & Ndou 2008; Ehlers 2006; Mulaudzi, Pengpid & Peltzer 2011). They are challenged, beyond the common stressors, with being exposed to death and dying patients, the stigma attached to the disease, fear of infection and lack of institutional material support (Delobelle *et al.* 2011; Hall 2004; Van Dyk 2007).

Generally, nurses’ work stress is positively related to burnout, work dissatisfaction, turnover intentions and eventual withdrawal from the job (arguably, in that order) (Delobelle *et al.* 2011; Hayes *et al.* 2012; Khamisa, Peltzer & Oldenburg 2013). Ultimately, loss of AIDS-dedicated and/or appropriately-trained personnel in the hospital care system is implicated in the poor quality of patient care (Kagee, Nothling & Coetzee 2012). The consequences are more pronounced in rural and public health facilities compared to private hospital settings (Pillay 2009).

The interrelationships between all factors playing a role in the experience of stress are complex, yet insufficiently studied (Khamisa *et al.* 2013).

### Problem statement

Studies are conducted on the stressful experiences of nurses caring for patients with AIDS (Hall 2004; Pillay 2009). Nonetheless, the study designs are limited in that they do not explore the complex relations between the concerned variables, leading to limited knowledge of the nurses’ experiences (Khamisa *et al.* 2013). Moreover, work–family (W-F) and family–work (F-W) conflicts and their psychological outcomes are studied extensively in the literature amongst various professionals in different settings, especially in developed countries. Yet, limited attention is given to health professionals working with patients who have AIDS in South Africa (Koekemoer & Mostert 2006). This study investigates the role of W-F and F-W conflicts, amongst the more common variables of the stress–strain model. Additionally, aside from nurses’ stress, variants of significant other and supervisor support, work and family satisfaction and turnover intentions are factored into the model to be tested.

#### Background

W-F and F-W conflicts are commonly defined as types of inter-role conflicts wherein some responsibilities from work and home domains are not compatible, exerting pressure on an individual and creating a conflict in which commitment to one set of pressure (family matters) increases difficulty in coping and complying with another set of pressure (work matters) (Boyar *et al.* 2003; Greenhaus & Beutell 1985). Studies conducted amongst nurses reveal that work overload, irregular work schedules and family role conflict are significant predictors of W-F and F-W conflicts which, in turn, are linked to consequences such as diminished life and work satisfaction, as well as turnover intentions (Alam & Mohammad 2010; Chirwa *et al.* 2009; Delobelle *et al.* 2011; Kaplan, Boshoff & Kellerman 1991; Kekana, du Rand & van Wyk 2007; Ramasodi 2010; Rittippant *et al.* 2011; Yildirim & Aycan 2008).

The relationship between W-F and F-W conflicts can be ameliorated or at least moderated by social support, especially the supervisor variant thereof. All forms of social support involve the exchange of resources such as materials, advice, help or understanding between at least two people with the aim of helping the support recipient (House 2001; Thoits 2011). Sources of support include co-workers and family members. A meta-analysis conducted by Byron (2005) linked family support to W-F conflict in both developed and non-developed countries. Delobelle *et al.* (2011) concluded that lack of support from management resulted in AIDS nurses in a hospital in Limpopo Province being prone to burnout reactions (see also Hall 2004; Mavhandu-Mudzusi, Netshandama & Davhana-Maselesele 2007).

#### Purpose of the study

The purpose of the study was to evaluate the capacity of W-F and F-W conflicts to predict work and life satisfaction, turnover intentions and the moderating role of supervisor and significant others’ support amongst nurses working with AIDS patients at government hospitals within the Capricorn and Mopani districts, Limpopo Province.

## Research design

### Research approach

The study utilised a cross-sectional design to collect data.

#### Population and sampling

The population of the study included all the nurses employed to care for AIDS patients in public hospitals within Mopani and Capricorn districts, Limpopo Province, South Africa. A convenience sampling procedure was utilised, whereby those nurses who were accessible and available at the AIDS wards in the respective hospitals were invited to participate in the study. Three hundred and nineteen (319) questionnaires were distributed to prospective participants. The final sample of the study comprised the 91 nurses who completed the questionnaires.

#### Data collection methods

The distribution of questionnaires was done by hand, with prospective participants being requested to volunteer and complete them. They were asked to provide their own dates when one of the researchers could collect the questionnaires. It took at least 45 minutes to complete the questionnaire when it was done through a face-to-face interview method. However, most participants asked to complete the questionnaires on their own, away from work, because of work pressures. At least 40 prospective participants requested replacement questionnaires since they misplaced or could not locate the first ones. The first author followed up on each of the participating nurses who were given a questionnaire to complete. She returned to the hospitals on the agreed-upon dates and left reminder messages with colleagues in the event that the nurses were not at work. She did this until she was satisfied that the particular participating nurses were eventually not going to complete the questionnaires. In the end, 91 questionnaires were fully completed, resulting in a 25% response rate.

#### Data collection instruments

Questionnaires which were distributed comprised the following: a demographic questionnaire; W-F and F-W conflict scales; turnover intentions scale; work satisfaction scale; family satisfaction scale; support behaviour inventory; significant other scale; and the nurses stress scale.

#### Demographic questionnaire

Demographic information was collected with a questionnaire that required respondents to state personal details such as age, ethnicity, gender and marital status. In addition, they were asked to declare whether they were associated with an HIV-focused group and whether they had attended an HIV-based workshop in the three months before participating in the present study.

#### Work-family and family-work conflict scales

W-F and F-W conflicts were measured using the Netemeyer, Boles and McMurrian (1996) scales of the same constructs. The five items on each scale were rated using a seven-point Likert-type response format, anchored from ‘strongly disagree’ (1) to ‘strongly agree’ (7). The present sample’s reliability level for both W-F and F-W conflict scales was α = 0.93 and α = 0.86, respectively.

#### Work satisfaction scale

Work satisfaction was assessed using the three-item general work satisfaction items from the Job Diagnostic Survey (Hennessy 2005). Using a 7-point Likert scale, ranging from ‘strongly disagree’ (1) to ‘strongly agree’ (7), participating nurses were asked to indicate the extent to which they agreed with the three work satisfaction items. The scale recorded a reliability of α = 0.78 in this study.

#### Family satisfaction scale

Family satisfaction was assessed using a shortened 5-item version of the quite established Brayfield and Rothe’s (1951, cited in Hennessy 2005) work satisfaction scale. The version used was a modification by Hennessy (2005). In the modified version, the word ‘work’ was replaced with the term ‘family life’. Extant W-F research has used measure modification of this nature (Aryee, Fields & Luk 1999; Hennessy 2005). Using a 5-point Likert scale, participants were asked to indicate the extent to which they were satisfied with the five family satisfaction items. The reliability level for the family satisfaction scale was α = 0.82 in this study.

#### Support behaviors inventory

The Support Behaviors Inventory (SBI) was developed by Brown in 1986. A short version of the SBI contains 11 items in which participants are asked to report the degree of satisfaction or dissatisfaction they experience with their partner’s support during pregnancy. For this study, items were adopted and modified so that participants report their satisfaction with their supervisor. It is based on a 6-point rating scale from ‘very dissatisfied’ (1) to ‘very satisfied’ (6). This study obtained a Cronbach’s alpha of 0.96.

#### Nurses stress scale

The Nurses Stress Scale was developed by Gray-Toft and Anderson (1981) as a measure of work-related stress for nurses working in hospital settings. Participant nurses in this study were provided a list of stressful situations common in hospital settings and were then instructed to state how often they found the situations to be stressful for them. Responses were done using a four-point response scale of ‘never’ (0), ‘occasionally’ (1), ‘frequently’ (2) and ‘very frequently’ (3). Although the scale has a total of seven subscales, this study used the full-scale scores of the shortened 34-item version to conduct analyses. In this study the reliability of the scale was α = 0.92.

#### Significant other scale

Significant other support was assessed with the 12-item Significant Other Scale, a measure originating from Zimet *et al.* (1988). The measure evaluates the amount of social support an individual is able to garner from significant others. The subtypes of support measured are family, friend and significant other. They are measured using a seven-step Likert-type scale ranging from ‘very strongly disagree’ to ‘very strongly agree’. For this study, the reliability level for the full-scale was α = 0.92; and for the significant other, friend and family subscales the Cronbach’s alpha reliability levels were 0.91, 0.90 and 0.78, respectively. This study used subscales of the measure.

#### Turnover intentions

‘Turnover intentions’, sometimes called intention to leave, is defined as an employee’s plan or aim of quitting a current job, looking forward to finding another one in the near future (Purani & Sahadev 2008). The three items of Purani and Sahadev’ s (2008) scale are rated on 5-point Likert scales, varying from ‘strongly disagree’ (1) to ‘strongly agree’ (5). The scale’s reliability in this study was α = 0.83.

#### Data collection procedure

The first author distributed 359 self-administered questionnaires (inclusive of 40 questionnaires sent to those nurses who requested a replacement of lost or misplaced copies), together with information sheets and consent forms to potential participants in all public hospitals, except for the Polokwane hospital, where permission for access was not received. The information sheet explained the purpose and significance of the study. It also detailed ethical considerations such as guaranteed anonymity, confidentiality and unconditional entitlement to withdraw participation. This was done to allow potential participants to make an informed decision about taking part in the study.

#### Data treatment

Data were coded, captured and analysed through the Statistical Package for Social Sciences, version 21 (SPSS 21.0) (IBM Corporation, Armonk, NY 2012). The main analysis involved the use of bivariate correlation analyses in order to investigate the associations between the main variables of the study. The results of the analysis were used to decide which variables were suitable for inclusion in further analysis. Hierarchical regression analysis was then used to predict work and family satisfaction and the turnover intentions variables. In each predictive analysis, the nurses’ stress was entered first as a control variable; W-F and F-W conflicts were entered second as independent variables; and family, significant other and supervisor supports were entered in the third step as moderating variables. Interaction terms, created from independent and moderator variables, were entered last.

## Results

### Characteristics of the sample

The ages of the participating nurses ranged from 22 to 59 years, with an average age of 36.85 (SD = 9.934). All participants were black African, 50.5% of them were single and the largest percentage (84.6%) of them were women. Participating nurses who were affiliated to an organisation that dealt with HIV accounted for 25.3%; and only 35.2% had attended an HIV workshop in the three months prior to data collection.

### Results of correlation analysis between all the study variables

The first step of the main analysis involved correlating all the study variables to determine their relationships. The gender and age of the participating nurses were not related to the rest of the variables. Nurses’ stress was positively associated with F-W and W-F conflicts (*p* ≤ 0.05), negatively related to work satisfaction (*p* ≤ 0.05) and its relationship with family satisfaction and all the support scales did not reach statistical significance (*p* > 0.05). F-W and W-F conflicts were negatively related to work satisfaction (*p* ≤ 0.05) and positively associated to turnover intentions (*p* ≤ 0.05), but the relationship with family satisfaction did not reach statistical significance (*p* > 0.05). F-W conflict was negatively associated to all forms of support measured (*p* ≤ 0.01) and W-F conflict was related only to supervisor support (*p* ≤ 0.01), also with a negative relationship. Work satisfaction was positively related to overall significant other (*p* ≤ 0.05) and supervisor (*p* ≤ 0.01) supports, whilst the relationship with social and family support did not reach statistical significance (*p* > 0.05). On the other hand, work satisfaction was negatively related to turnover intentions (*p* ≤ 0.01) and was not related to family satisfaction (*p* > 0.05). Family satisfaction was positively related to all the varieties of significant other support (*p* ≤ 0.05), but its relationship to supervisor support did not reach statistical significance (*p* > 0.05). Turnover intentions were not related to any of the social support scales (*p* > 0.05) or family satisfaction (*p* > 0.05) (Table 1).

**TABLE 1:** Correlation analyses of all study variables (*N* = 91).

Variable	1	2	3	4	5	6	7	8	9	10	11
1. Gender†	–	–	–	–	–	–	–	–	–	–	–
2. Age	0.200	–	–	–	–	–	–	–	–	–	–
3. Nurses stress	0.203	0.123	–	–	–	–	–	–	–	–	–
4. F-W conflict	−0.025	−0.142	0.210*	–	–	–	–	–	–	–	–
5. W-F conflict	0.021	0.035	0.291**	0.428**	–	–	–	–	–	–	–
6. Work satisfaction	0.146	0.053	−0.248*	−0.467**	−0.292**	–	–	–	–	–	–
7. Family satisfaction	0.043	−0.089	0.024	−0.133	−0.043	0.189	–	–	–	–	–
8. Turnover intentions	−0.143	−0.163	0.192	0.396**	0.442**	−0.584**	−0.131	–	–	–	–
9. Significant other support	0.010	−0.081	−0.062	−0.452**	−0.146	0.227*	0.398**	−0.102	–	–	–
10. Friend support	−0.048	−0.071	−0.168	−0.453**	−0.173	0.205	0.328**	−0.076	0.855**	–	–
11. Family support	0.030	−0.095	−0.062	−0.367**	−0.075	0.16	0.314**	−0.093	0.919**	0.764**	–
12. Supervisor support	−0.181	−0.158	−0.089	−0.398**	−0.367**	0.337**	0.151	−0.193	0.300**	0.264*	0.232*

†, Spearman rho coefficient; F-W, family-work; W-F, work-family.

*p ≤ 0.05, **p ≤ 0.01

### The prediction of work and family satisfaction, and turnover intentions

#### Direct effect of work-family and family-work conflicts

The results from the analysis supported the hypothesis that F-W conflict will predict work satisfaction amongst nurses (*p* ≤ 0.001), whilst the hypothesis that W-F conflict will predict work satisfaction was not supported (*p* > 0.05) (Table 2a, model 2). In addition, W-F and F-W conflicts were not related to family satisfaction (*p* > 0.05), meaning that the results failed to confirm the hypothesis that W-F and F-W conflicts will predict family satisfaction (Table 2b, model 2). The hypothesis that W-F conflict will predict turnover intentions amongst nurses was supported (*p* ≤ 0.05), but the results failed to support the hypothesis that F-W conflict will predict turnover intentions (*p* > 0.05) (Table 2c, model 2).

**TABLE 2(a):** Results of hierarchical regression analyses for the prediction of work satisfaction.

Variables	Model 1	Model 2	Model 3	Model 4
**Step 1: control variable**				
Nurses stress scale	−0.274*	−0.178†	−0.172†	−0.201†
**Step 2: independent variable**				
F-W conflict	–	−0.405***	−0.356**	0.349***
**Step 3: moderator**				
SS x W-F conflict	–	–	0.210*	–
**Step 4: interaction terms**				
SS x W-F conflict	–	–	–	0.268**
SOS x W-F conflict	–	–	–	−0.249**
*R*^2^	0.075	0.2413	0.272	0.33
Adjusted *R*^2^	0.064	0.211	0.243	0.294
*F*	6.437**	11.677***	9.582***	9.348***
∆*R*^2^	–	0.155	0.041	0.058

F-W, family-work; W-F, work-family; SS, supervisor support; SOS, significant other support.

†*p* < 0.10; *p < 0.05; **p < 0.01; ***p < 0.001

**TABLE 2(b):** Results of hierarchical regression analyses for the prediction of family satisfaction.

Variables	Model 1	Model 2
**Step 1: control variable **		
SOS	0.323**	0.534***
**Step 2: independent variable**		
SOS x W-F conflict	–	−0.372**
*R*^2^	0.104	0.198
Adjusted *R*^2^	0.093	0.178
*F*	9.200**	9.648***
∆*R*^2^	–	0.094

W-F, work-family; SS, supervisor support; SOS, significant other support.

†, *p* < 0.10; *p < 0.05; **p < 0.01; ***p < 0.001

**TABLE 2(c):** Results of hierarchical regression analyses for the prediction of turnover intentions.

Variables	Model 1
**Step 1: independent variable**	
W-F conflict	489*
*R*^2^	0.240
Adjusted *R*^2^	0.230
*F*	24.877*
∆*R*^2^	0.240

W-F, work-family.

* *p* < 0.001

#### Family support as moderating variable

Regression analysis showed that family support was not a moderator of the relationship between W-F or F-W conflicts and work and family satisfaction and turnover intentions, respectively (*p* > 0.05) (Tables 2a–c). Thus, the hypotheses specifying moderation were not supported. In the first place, when work and family satisfaction and turnover intentions were each regressed on W-F or F-W conflicts, no association was established, meaning that there was no effect to moderate.

#### Support from significant others as moderating variable

The results of regression analysis showed that none of the varieties of significant other support moderates the relationship between reports of W-F and F-W conflicts from nurses and any of the dependent variables, namely, work and family satisfaction and turnover intentions (*p* > 0.05) (Tables 2a–c). However, significant other support had a direct effect on family satisfaction, impacting it positively (*p* ≤ 0.01) (Table 2b, model 1). Also, the results show that even if support from significant others did not moderate W-F and F-W conflicts, the interaction between significant other support and W-F conflict was negatively related to work satisfaction and the relationship was statistically significant (*p* ≤ 0.01) (Table 2a, model 4). The interaction between significant other support and W-F conflict was also significantly negatively related to family satisfaction (*p* ≤ 0.01) (Table 2b, model 2).

#### Support from the nurses’ supervisors as moderating variable

A finding of this study indicates that supervisor support moderated only experiences of W-F conflict and work satisfaction (*p* ≤ 0.05) (Table 2a, model 3). Other dependent variables such as family satisfaction and turnover intentions were not moderated. Again, the interaction between supervisor support and W-F conflict was negatively related to work satisfaction and the relationship was statistically significant (*p* ≤ 0.01) (Table 2a, model 4).

## Ethical considerations

Ethical clearance (Ethical clearance number: TREC2010/087-121) was obtained from the Research and Ethics Committee of the University of Limpopo. Permission to access potential participants was given by the Limpopo Department of Health and Social Development and relevant managers in each of the sampled public hospitals within the Capricorn and Mopani districts.

## Discussion

The present study used hierarchical regression analysis to explore the existence of W-F and F-W conflicts amongst nurses working with patients who have AIDS. The main objective of the study was to find out if the conflict types lead to work and family dissatisfaction and turnover intentions amongst nurses in hospitals within the Limpopo Province, South Africa. In addition, the study examined the moderator role of family support and social support from supervisor and significant other in relation to W-F and F-W conflicts and its outcomes.

It is important to highlight certain issues pertaining to the interpretation of the results. To begin with, the return rate of 25% in this study should be taken into account when interpreting the results. Although the magnitude of the return rate itself is not unusual for a social science survey, its meaning is important. The participants may have been a self-selected group, indicating that their views may be unique. This means that care must be exercised in generalising the results.

### Association of work and family conflicts to dependent variables

A finding that F-W conflict predicted only work satisfaction is consistent with outcomes from Mesmer-Magnus and Viswesvaran (2005), who concluded that family aspects interfering with work tasks (that is, F-W conflict), but not W-F conflict, are negatively related to work performance and attitudes. These findings are similar to what was established by Zhao, Qu and Ghiselli (2011). The findings are nonetheless not consistent with some previous studies which indicate that, in fact, W-F conflict is related to lower work and family satisfaction amongst employees. For instance, Cortese, Colombo and Ghislieri (2010) found that W-F conflict in health organisations contributed toward decreased work satisfaction amongst 351 North Italian hospital nurses. More recently, Gao *et al.* (2012) also found that W-F conflict was negatively related to work satisfaction. Moreover, Almalki, Fitzgerald and Clark (2012) observed that dissatisfaction with work life amongst nurses in the Jazan region of Saudi Arabia is a result of their inability to balance work and family needs and supervision practices, amongst others.

In addition, W-F conflict predicted only turnover intentions amongst the present sample. This finding added evidence to previous literature confirming a relationship between the two variables. For example, Farquharson *et al.* (2012) found that W-F conflict was a significant predictor of turnover intentions and work satisfaction amongst 152 nurses working in a healthcare telephone advice service.

The inconsistency of the present results when compared to existing findings in and outside South Africa in relation to W-F conflict, work and family satisfaction is surprising. This, however, could be linked to the cultural background of the present sample. Studies argue that experiences of W-F conflict are culture specific and not global (Powell, Francesco & Ling 2009; Trefalt *et al.* 2013). Contrasting culture styles have been identified, namely, individualistic and collectivistic orientations. One of their distinguishing characteristics is their particular attitudes toward work and family. South Africans, particularly those of African descent, are part of a collectivistic culture which worries less about work activities interfering with family activities (Allik & McCrae 2004; Gamor, Amissah & Boakye 2014; Okonkwo 2014; Spector *et al.* 2007). They view work as serving the needs of the family; therefore, individuals who devote time and effort in it are supported by their respective families (but see Allen *et al.* 2015).

### The moderating effect of family support

Family support failed to moderate the relationship between nurses’ reports of W-F and F-W conflicts and experiences of work and family satisfaction and turnover intentions. This finding is inconsistent with previous research. For instance, Ford, Heinen and Langkamer (2007) established that family support reduced experiences of W-F and F-W conflicts. Results on the impact of family support as a moderator will be clarified more below, in the context of significant other and supervisor supports. This is because only a few studies focus specifically on this type of support as a moderator variable (Rittippant *et al.* 2011). Spouse support has been considered to be the primary source of support in the family domain (Cinamon 2009; Cinamon & Rich 2005).

Although support from significant others did not moderate the relationship between reports of W-F and F-W conflicts from nurses and any of the dependent variables, such as work and family satisfaction and turnover intentions, it had a direct effect on family satisfaction. Therefore, the hypothesis was partially supported and was consistent with prior findings that concluded that social support from spouses and significant others reduced W-F conflict (Cinamon 2009; Cinamon & Rich 2005; Huffman, Casper & Payne 2014). Furthermore, Patel *et al.* (2008) ‘explored the impact of work on family functioning, its relationship to work satisfaction and the role of spousal support in a group of 80 female nurses working in a government hospital’ in South Africa. Their study revealed that there was a negative relationship between work satisfaction, spousal support and W-F conflict.

### The moderating effect of support from a supervisor

The hypothesis for moderation was supported in part because supervisor support moderated reports of W-F conflict and experiences of work satisfaction. In addition, the interaction between supervisor support and W-F conflict was negatively related to work satisfaction. This finding supports studies such as that of Kossek *et al.* (2011), and Anderson, Coffey and Byerly (2002), who stated that supervisor support and all employee outcomes were directly related, whilst negative career consequences were related to lower work satisfaction and higher turnover intentions.

Supervisor support failed to moderate experiences of family dissatisfaction and turnover intentions in this sample. This finding is surprising, since supervisor support has sometimes been found to serve as a moderator. Muhammad and Hamdy (2005) reported that social support from supervisor and colleagues moderated the relationship between burnout and turnover intentions. However, there are studies supporting lack of moderating effect of supervisor support (Ahmed, Muddasar & Perviaz 2012; Frone, Russell & Cooper 1992; Yildirim & Aycan 2008). Yildirim and Aycan (2008) observed that supervisor support only has a direct rather than a moderating effect. Indeed, results of correlation analysis in the present study demonstrate the direct relationship between supervisor support and both W-F and F-W conflicts. It is of note that House’s seminal text cautioned more than 30 years ago that cross-sectional designs are not the best platform to demonstrate the moderating role of social support (House 1981).

## Limitations of the study

This study provides an important comprehensive insight on the experiences and consequences of W-F and F-W conflicts of nurses working with patients living with AIDS in the Limpopo Province; and has a potential to contribute to the limited W-F and F-W conflicts research literature in South Africa, as well as the nursing practice by identifying some of the factors leading to W-F and F-W conflicts. Nevertheless, there are limitations that are important to consider when interpreting findings of this study. Firstly, the study had a relatively small sample size (*N* = 91), spread across a relatively wide geographic area. Therefore, caution must be exercised when generalising the findings to all nurses, particularly because of different environmental factors and organisational challenges unique to each hospital which may impact nurses’ satisfaction with their jobs and families and experiences of W-F and F-W conflicts and turnover intentions.

Secondly, it is likely that most of the participants provided a subjective perception when responding to the self-administered questionnaire which they completed in their own spare times. None of the primary variables of the study were supplemented with objective data gathering methods. Moreover, a cross-sectional design limited the researcher’s ability to draw causal inferences amongst the study variables.

## Recommendations

In future, more studies on W-F and F-W conflicts need to be conducted within the South African nursing environment. Findings can be compared to similar international studies. More intervention programmes can be adopted in order to help nurses identify and cope with W-F and F-W conflict issues affecting their family and work satisfaction and performance. Furthermore, qualitative studies are required in order to explore and describe the inter-role experiences of nurses in Limpopo.

## Conclusion

Findings from the study provided limited support of the F-W and W-F conflicts impacts, showing that F-W conflict predicted work satisfaction whilst W-F conflict predicted the nurses’ turnover intentions. It was argued in this study that experiences of W-F and F-W conflicts are not global but culture specific; therefore, this could have affected the findings of the study. Furthermore, the interaction between supervisor support and W-F conflict had a negative relationship with work satisfaction.

Although some of the hypotheses of the study – such as W-F’s prediction of work satisfaction, the prediction of family satisfaction by W-F and F-W conflicts and the moderation of the relationship between W-F and F-W conflicts and turnover intentions, work and family satisfaction – were not supported, findings from this study have significant implications for family and nursing institutions by contributing to the limited literature on W-F and F-W conflicts in South Africa. In order to reduce nurses’ experiences of W-F and F-W conflicts, working conditions such as staff shortages should be improved by ensuring that more nurses are trained and recruited to work in units that deal with patients living with AIDS. Interventions aimed at improving nurses’ family and work support, as well as issues relating to family and work balance should be implemented.

## References

[CIT0001] Ahmed M., Muddasar M. & Perviaz S., 2012, ‘The impact of work–family conflict and pay on employee job satisfaction with the moderating effect of perceived supervisor support in Pakistan banking sector’, *Global Journal of Management* & *Business Research* 12(6), 8.

[CIT0002] Alam M.M. & Mohammad J.F., 2010, ‘Level of job satisfaction and intent to leave among Malaysian nurses’, *Business Intelligence Journal* 3(1), 123–137.

[CIT0003] Allen T.D., French K.A., Dumani S. & Shockley K.M., 2015, ‘Meta-analysis of work–family conflict mean differences: Does national context matter?’, *Journal of Vocational Behavior* 90, 90–100. 10.1016/j.jvb.2015.07.006

[CIT0004] Allik J. & McCrae R.R., 2004, ‘Toward a geography of personality traits: Patterns of profiles across 36 cultures’, *Journal of Cross-Cultural Psychology* 35(1), 13–28. 10.1177/0022022103260382

[CIT0005] Almalki M.J., Fitzgerald G. & Clark M., 2012, ‘Quality of work life among primary health care nurses in the Jazan region, Saudi Arabia: A cross-sectional study’, *Human Resources for Health* 10, 30. PMID: , 10.1186/1478-4491-10-30 22971150 PMC3543175

[CIT0006] Anderson S.E., Coffey B.S. & Byerly R.T., 2002, ‘Formal organizational initiatives and informal workplace practices: Links to work-family conflict and job-related outcomes’, *Journal of Management* 28(6), 787–810. 10.1177/014920630202800605

[CIT0007] Aryee S., Fields D. & Luk V., 1999, ‘A cross-cultural test of a model of the work-family interface’, *Journal of Management* 25(4), 491–511. 10.1177/014920639902500402

[CIT0008] Boyar S.L., Maertz C.P., Pearson A.W. & Keough S., 2003, ‘Work–family conflict: A model of linkages between work and family domain variables and turnover intentions’, *Journal of Managerial Issues* 15(2), 175–190.

[CIT0009] Brayfield A.H. & Rothe H.F., 1951, ‘An index of job satisfaction’, *Journal of Applied Psychology* 35(5), 307–311. 10.1037/h0055617

[CIT0010] Brown M.A., 1986, ‘Social support during pregnancy: A unidimensional or multidimensional construct?’, Journal of Nursing Research 35(1), 4–9. PMID: . 3632846

[CIT0011] Byron K., 2005, ‘A meta-analytic review of work–family conflict and its antecedents’, *Journal of Vocational Behavior* 67(2), 169–198. 10.1016/j.jvb.2004.08.009

[CIT0012] Chirwa M.L., Greef M., Kohi T.W., Naidoo J.R., Makoae L.N., Dlamini P.S. et al., 2009, ‘HIV stigma and nurse job satisfaction in five African countries’, *Journal of the Association of Nurses in AIDS Care* 20(1), 14–21. PMID: , 10.1016/j.jana.2008.10.001 19118767 PMC2743864

[CIT0013] Cinamon R.G., 2009, ‘Role salience, social support, and work–family conflict among Jewish and Arab female teachers in Israel’, *Journal of Career Development* 36(2), 139–158. 10.1177/0894845309345849

[CIT0014] Cinamon R.G. & Rich Y., 2005, ‘Work–family conflict among female teachers’, *Teaching and Teacher Education* 21(4), 365–378. 10.1016/j.tate.2004.06.009

[CIT0015] Coetzee S.K., Klopper H.C., Ellis S.M. & Aiken L.H., 2013, ‘A tale of two systems – Nurses practice environment, well being, perceived quality of care and patient safety in private and public hospitals in South Africa: A questionnaire survey’, *International Journal of Nursing Studies* 50(2), 162–173. PMID: , 10.1016/j.ijnurstu.2012.11.002 23218020

[CIT0016] Cortese C.G., Colombo L. & Ghislieri C., 2010, ‘Determinants of nurses’ job satisfaction: The role of work–family conflict, job demand, emotional charge and social support’, *Journal of Nursing Management* 18(1), 35–43. PMID: , 10.1111/j.1365-2834.2009.01064.x 20465727

[CIT0017] Delobelle P., Rawlinson J.L., Ntuli S., Malatsi I., Decock R. & Depoorter A.M., 2011, ‘Job satisfaction and turnover intent of primary healthcare nurses in rural South Africa: A questionnaire survey’, *Journal of Advanced Nursing* 67(2), 371–383. PMID: , 10.1111/j.1365-2648.2010.05496.x 21044134

[CIT0018] De Villiers L.D. & Ndou N.D., 2008, ‘South African professional nurses’ experiences of caring for HIV/AIDS patients’, *Africa Journal of Nursing and Midwifery* 10(1), 5–21.

[CIT0019] Ehlers V.J., 2006, ‘Challenges nurses face in coping with the HIV/AIDS pandemic in Africa’, International Journal of Nursing Studies 43(6), 657–662. PMID: 16436278. 16436278 10.1016/j.ijnurstu.2005.11.009

[CIT0020] Farquharson B., Allan J., Johnston D., Johnston M., Choudhary C. & Jones M., 2012, ‘Stress amongst nurses working in a healthcare telephone-advice service: Relationship with job satisfaction, intention to leave, sickness absence, and performance’, *Journal of Advanced Nursing* 68(7), 1624–1635. PMID: , 10.1111/j.1365-2648.2012.06006.x 22621255

[CIT0021] Ford M.T., Heinen B.A. & Langkamer K.L., 2007, ‘Work and family satisfaction and conflict: A meta-analysis of cross-domain relations’, *Journal of Applied Psychology* 92(1), 57–80. PMID: , 10.1037/0021-9010.92.1.57 17227151

[CIT0022] Frone M.R., Russell M. & Cooper M.L., 1992, ‘Antecedents and outcomes of work-family conflict: Testing a model of the work-family interface’, *Journal of Applied Psychology*, 77(1), 65–78. PMID: , 10.1037//0021-9010.77.1.65 1556042

[CIT0023] Gamor E., Amissah E.F. & Boakye K.A.A., 2014, ‘Work–family conflict among hotel employees in Sekondi-Takoradi Metropolis, Ghana’, *Tourism Management Perspectives* 12, 1–8. 10.1016/j.tmp.2014.06.001

[CIT0024] Gao Y., Shi J., Niu Q. & Wang L., 2012, ‘Work–family conflict and job satisfaction: Emotional intelligence as a moderator’, *Stress and Health* 29(3), 222–228. PMID: , 10.1002/smi.2451 23015466

[CIT0025] Gray-Toft P. & Anderson J.G., 1981, ‘Stress among hospital nursing staff: Its causes and effects’, *Social Science* & *Medicine.* *Part A: Medical Sociology* 15(5), 639–647. PMID: , 10.1016/0271-7123(81)90087-0 6976625

[CIT0026] Greenhaus J.H. & Beutell N.J., 1985, ‘Sources of conflict between work and family roles’, *The Academy of Management Review* 10(1), 78–88. 10.5465/AMR.1985.4277352

[CIT0027] Hall E.J., 2004, *The challenges HIV/AIDS poses to nurses in their work environment*, Employment & Economic Policy Research, Human Sciences Research Council, Pretoria.

[CIT0028] Hayes L.J., O’Brien-Pallas L., Duffield C., Shamian J., Buchan J., Hughes F. et al., 2012, ‘Nurse turnover: A literature review – An update’, *International Journal of Nursing Studies* 49(7), 887–905. PMID: , 10.1016/j.ijnurstu.2011.10.001 22019402

[CIT0029] Hennessy D.K., 2005, *Work*–*family conflict self-efficacy: A scale validation study*, Masters’ thesis, Dept. of Counseling and Personnel Services, University of Maryland.

[CIT0030] House J.S., 1981, *Work stress and social support*, Addison-Wesley Longman, Reading, MA.

[CIT0031] House J.S., 2001, ‘Social isolation kills, but how and why?’, *Psychosomatic Medicine* 63(2), 273–274. PMID: , 10.1097/00006842-200103000-00011 11292275

[CIT0032] Huffman A.H., Casper W.J. & Payne S.C., 2014, ‘How does spouse career support relate to employee turnover? Work interfering with family and job satisfaction as mediators’, *Journal of Organizational Behavior* 35(2), 194–212. 10.1002/job.1862

[CIT0033] Kagee A., Nothling J. & Coetzee B., 2012, ‘The perspectives of users of antiretroviral therapy on structural barriers to adherence in South Africa’, *South African Family Practice* 54(6), 540–544. 10.1080/20786204.2012.10874289

[CIT0034] Kaplan R.A., Boshoff A.B. & Kellerman A.M., 1991, ‘Job involvement and job satisfaction of South African nurses compared with other professions’, Curationis 14(1), 3–7. PMID: 1845612. 1845612 10.4102/curationis.v14i1.309

[CIT0035] Kekana H.P., du Rand E.A. & van Wyk N.C., 2007, ‘Job satisfaction of registered nurses in a community hospital in the Limpopo Province in South Africa’, *Curationis* 30(2), 24–35. PMID: , 10.4102/curationis.v30i2.1068 17703820

[CIT0036] Khamisa N., Peltzer K. & Oldenburg B., 2013, ‘Burnout in relation to specific contributing factors and health outcomes among nurses: A systematic review’, *International Journal of Environmental Research and Public Health* 10(6), 2214–2240. PMID: , 10.3390/ijerph10062214 23727902 PMC3717733

[CIT0037] Koekemoer F.E. & Mostert K., 2006, ‘Job characteristics, burnout and negative work–home interference in a nursing environment’, *South African Journal of Industrial Psychology* 32(3), 79–86.

[CIT0038] Kossek E.E., Pichler S., Bodner T. & Hammer L.B., 2011, ‘Workplace social support and work–family conflict: A meta-analysis clarifying the influence of general and work–family-specific supervisor and organizational support’, Personnel Psychology 64(2), 289–313. PMID: 21691415. 21691415 10.1111/j.1744-6570.2011.01211.x PMC3116443

[CIT0039] Mavhandu-Mudzusi A.H., Netshandama V.O. & Davhana-Maselesele M., 2007, ‘Nurses’ experiences of delivering voluntary counselling and testing services for people with HIV/AIDS in the Vhembe District, Limpopo Province, South Africa’, Nursing & Health Sciences 9(4), 254–262. PMID: 17958674. 10.1111/j.1442-2018.2007.00341.x 17958674

[CIT0040] Mesmer-Magnus J.R. & Viswesvaran C., 2005, ‘Convergence between measures of work-to-family and family-to-work conflict: A meta-analytic examination’, *Journal of Vocational Behavior* 67(2), 215–232. 10.1016/j.jvb.2004.05.004

[CIT0041] Muhammad A.H. & Hamdy H.I., 2005, ‘Burnout, supervisory support, and work outcomes: A study from an Arabic cultural perspective’, *International Journal of Commerce and Management* 15(3/4), 230–242. 10.1108/10569210580000199

[CIT0042] Mulaudzi M.V., Pengpid S. & Peltzer K., 2011, ‘Nurses’ knowledge, attitudes, and coping related to HIV and AIDS in a rural hospital in South Africa’, *Studies on Ethno-Medicine* 5(1), 25–32.

[CIT0043] Netemeyer R.G., Boles J.S. & McMurrian R., 1996, ‘Development and validation of work–family conflict and family–work conflict scales’, *Journal of Applied Psychology* 81(4), 400–410. 10.1037/0021-9010.81.4.400

[CIT0044] Okonkwo E., 2014, ‘Female nurses experiencing family strain interference with work: Spousal support and number of children impacts’, *Gender* & *Behaviour* 12(1), 6182–6188.

[CIT0045] Patel C.J., Beekhan A., Paruk Z. & Ramgoon S., 2008, ‘Work–family conflict, job satisfaction and spousal support: An exploratory study of nurses’ experience’, Curationis 31(1), 38–44. PMID: 18592947. 18592947 10.4102/curationis.v31i1.906

[CIT0046] Pillay R., 2009, ‘Work satisfaction of professional nurses in South Africa: A comparative analysis of the public and private sectors’, *Human Resources for Health* 7, 15. PMID: , 10.1186/1478-4491-7-15 19232120 PMC2650673

[CIT0047] Powell G.N., Francesco A.M. & Ling Y., 2009, ‘Toward culture-sensitive theories of the work–family interface’, *Journal of Organizational Behavior* 30(5), 597–616. 10.1002/job.568

[CIT0048] Purani K. & Sahadev S., 2008, ‘The moderating role of industrial experience in the job satisfaction, intention to leave relationship: An empirical study among salesmen in India’, *Journal of Business* & *Industrial Marketing* 23(7), 475–485. 10.1108/08858620810901239

[CIT0049] Ramasodi J.M.B., 2010, ‘Factors influencing job satisfaction among healthcare professionals at South Rand hospital’, Masters’ thesis, School of Public Health, University of Limpopo.

[CIT0050] Rittippant N., Tongkong J., Thamma-Apiroam S. & Mingariyamark S., 2011, ‘Work–family conflict: An investigation of healthcare professionals in Thailand’, *International Proceedings of Economic Development* & *Research* 8, 64–68.

[CIT0051] Spector P.E., Allen T.D., Poelmans S.A.Y., Lapierre L.M., Cooper C.L., O’Driscoll M. et al., 2007, ‘Cross-national differences in relationships of work demands, job satisfaction, and turnover intentions with work–family conflict’, *Personnel Psychology* 60(4), 805–835. 10.1111/j.1744-6570.2007.00092.x

[CIT0052] Thoits P.A., 2011, ‘Mechanisms linking social ties and support to physical and mental health’, *Journal of Health and Social Behavior* 52(2), 145–161. PMID: , 10.1177/0022146510395592 21673143

[CIT0053] Trefalt Š., Dmovšek M., Svetina-Nabergoj A. & Adlešič R.V., 2013, ‘Work-life experiences in rapidly changing national contexts: Structural misalignment, comparisons and choice overload as explanatory mechanisms’, *European Management Journal* 31(5), 448–463. 10.1016/j.emj.2013.04.006

[CIT0054] van der Colff J.J., 2005, ‘Work-related well-being of registered nurses in South Africa’, Doctoral thesis, Dept. of Industrial Psychology, University of North-West.

[CIT0055] van Dyk A.C., 2007, ‘Occupational stress experienced by caregivers working in the HIV/AIDS field in South Africa’, *African Journal of AIDS Research* 6(1), 49–66. PMID: , 10.2989/16085900709490399 25875345

[CIT0056] Yildirim D. & Aycan Z., 2008, ‘Nurses’ work demands and work–family conflict: A questionnaire survey’, *International Journal of Nursing Studies* 45(9), 1366–1378. PMID: , 10.1016/j.ijnurstu.2007.10.010 18262529

[CIT0057] Zhao X., Qu H. & Ghiselli R., 2011, ‘Examining the relationship of work–family conflict to job and life satisfaction: A case of hotel sales managers’, *International Journal of Hospitality Management* 30(1), 46–54. 10.1016/j.ijhm.2010.04.010

[CIT0058] Zimet G.D., Dahlem N.W., Zimet S.G. & Farley G.K., 1988, ‘The Multidimensional scale of perceived social support’, *Journal of Personality Assessment* 52(1), 30–41. 10.1207/s15327752jpa5201_2 2280326

